# TR Locus Annotation and Characteristics of *Rhinolophus ferrumequinum*

**DOI:** 10.3389/fimmu.2021.741408

**Published:** 2021-09-30

**Authors:** Hao Zhou, Long Ma, Longyu Liu, Xinsheng Yao

**Affiliations:** Department of Immunology, Center of Immunomolecular Engineering, Innovation & Practice Base for Graduate Students Education, Zunyi Medical University, Zunyi, China

**Keywords:** TR loci, annotation, IMGT, bat, T-cell receptor, immunogenomics

## Abstract

T-cell antigen receptors (TRs) in vertebrates can be divided into αβ or γδ, encoded by TRA/D, TRG, or TRB loci. TRs play a central role in mammal cellular immunity, which occurs by rearrangement of V, D, J, and C genes in the loci. The bat is the only mammal with flying ability and is considered the main host of zoonotic viruses, an important public health concern. However, at present, little is known about the composition of bat TR genes. Based on the whole genome sequence of the greater horseshoe bat (*Rhinolophus ferrumequinum*) and referring to the TR/IG annotation rules formulated by the international ImMunoGeneTics information system (IMGT), we present a complete annotation of TRA/D, TRG, and TRB loci of *R. ferrumequinum*. A total of 128 V segments, three D segments, 85 J segments, and 6 C segments were annotated and compared with other known mammalian data. The characteristics of the TR locus and germline genes of *R. ferrumequinum* are analyzed.

## 1 Introduction

Vertebrate T cells participate in adaptive immune responses *via* their diversified T-cell antigen receptors (TRs) on the surface of cells. TRs are composed of two polymorphic chains, which can be divided into two types: αβ and γδ polypeptide chains encoded by four loci, TRA, TRD, TRB, and TRG. Each polypeptide chain contains a variable region and a constant region, rearranged by germline V (variable) gene and/or D (diversity) gene and J (joining) gene (1, 2). The TR recombination process is conducted by the product of recombination activating genes (RAG-1/RAG-2) by making double-strand breaks at the recombination signal sequence and then shearing and inserting to rearrange V(D)J genes (3). Immunoglobulin (IG) and TR loci contain hundreds of germline genes. For example, humans have 608–665 B-cell receptor (BCR) and T-cell receptor (TCR) genes, and mice contain more than 800 BCR and TCR genes, which can be divided into functional genes, open reading frames (ORFs), and pseudogenes, according to functionality (4).

The annotation of TR and IG loci is challenging because germline genes do not have the classical intron/exon structure that can be detected by standard annotation software. Furthermore, most of the sequences are very short; J gene is between 40 and 60 bp, and D gene is only approximately 10 bp. The international ImMunoGeneTics information system (IMGT; http://www.imgt.org/) is the authoritative database for immunogenetics and immunoinformatics, which describes the accurate annotation rules for TR and IG genes in detail. In the process of locus annotation of TR or IG, the identification of V, D, J, and C genes is the most important part. This must then be followed by a complete description of each gene. For example, a complete V gene segment includes L-part (L-part1+L-part2)+V-region+V-RSS. Next is the functional determination of germline genes, which can be divided into three types according to IMGT: 1) functional gene (functional, F) means that there is an ORF and no defect of stop codon and splice site. Based on the severity of the identified defect, it can be defined as 2) ORF or 3) P (pseudogene, P). The final step is to classify the group or subgroup according to the nucleotide similarity and the location of the cluster (5–7).

Vertebrates have four TR loci, which, to date, have been completely or partially annotated in humans, mice, cattle, and some other species (http://www.imgt.org/IMGTrepertoire/LocusGenes/#C). The structural characteristics of different loci have also been summarized. For species after teleost, TRA, and TRD loci are in the same loci. TRA and TRD loci share part of V gene but have independent J and C genes. The mammalian TRD locus is usually located downstream of TRAV gene. Upstream of TRAJ gene, there is always a TRDV gene with the opposite transcription direction downstream of TRDC gene (8). A large number of VHδ genes were found in TRA/D loci of *Xenopus* and platypus, which were highly homologous to their own IGHV. These VHδ genes could rearrange with Dδ, Jδ, and Cδ genes (9, 10). In addition, a fifth TCRμ locus was found in monotremes and marsupials, and Vμ gene and VHδ of platypus owned the clearest evolutionary association. Researchers hypothesized that TCRμ locus originated from the recombination of V gene contributed by IgH locus and D, J, and C genes contributed by TRD locus (11–14). TRG locus usually has two structures: one is the typical form of V–J–C translocon, where the replication occurs in J–C clusters, such as in humans, rabbits, and possums. The other comprises V–J–C clusters formed in multiples due to genome duplication, such as in cattle, dogs, and mice (15). The structure of TRB locus in mammals is relatively conservative. Semi-clusters are a common feature, and upstream usually contains multiple V genes with varying numbers, and several D–J–C clusters distributed downstream. The D–J–C cluster increases the size of the germline gene pool that can be used for recombination (16).

There are more than 1,400 different bats, which together account for 20% of all mammals and occur in six continents (17, 18). Bat (Chiroptera) consists of two suborders, based on morphological, molecular, and fossil evidence: Yinpterochiroptera (which comprises megabats and several families of microbats) and Yangochiroptera (comprises all remaining microbats families) (19–22). Bats are host to many viruses, including many virulent ones, but studies have indicated that these viruses typically do not cause clinical symptoms for the bats (23–25). Although many immune-related genes have been identified in some bat species, lack of experimental models and related reagents have meant that research on the adaptive immunity of bats is limited (22). Major mammalian antibody subclasses have been detected in bats, including IgA, IgE, IgM, and IgG (26, 27). Early studies reported that the scale level and duration of antibody reaction of bats to antigens might be lower in virus-infected bats, but it is still unclear the specific role of antibodies in virus infection. At the genome level, bats seem to have more BCR germline genes than humans, which may provide more antigen specificity for the naive BCR. Meanwhile, no somatic hypermutation was found in the small brown bat, which also indicates that bats may rely more on germline pools to respond to infection (28, 29).

At present, the genomes and transcriptomes of at least 44 species of bats are available in the database (30, 31). However, due to the incomplete sequencing and annotation of these bats’ genomes and transcriptomes, the systematic and complete annotation of bats’ whole genome has not been completed. In particular, the annotation of bats’ immune genes such as TCR and BCR is currently lacking. The degree of genome analysis at the chromosome level only has three bats: *Rhinolophus ferrumequinum* (greater horseshoe bat), *Phyllostomus discolor* (pale spear-nosed bat), and *Pipistrellus pipistrellus* (common pipistrelle). The greater horseshoe bat is an insectivorous bat species with a very wide distribution in the Palearctic, occurring in northern Africa, southern Europe, and Asia (32, 33). We evaluated the integrity of genome and the difficulty of obtaining samples. Using the annotation rules of IMGT-ONTOLOGY, we annotated TRA/D, TRB, and TRG of the greater horseshoe bat (*R. ferrumequinum*) completely; and we compared the annotated genes with those of humans, mice, pigs, dogs, cattle, and other species in the database.

## 2 Materials and Methods

### 2.1 Annotation of the T-Cell Antigen Receptor Locus of *Rhinolophus ferrumequinum*

Based on the whole genome sequence (GCA_004115265.3) shared by the National Center for Biotechnology Information (NCBI) and submitted by the Vertebrate Genomes Project to whole genome shotgun sequencing, the genome has 28 chromosomes, with a total length of 2,075.77 Mb. We were able to complete the current analysis because of the high quality and coverage of this genome. **Supplementary Table 1** provides additional information of whole genome sequence of the greater horseshoe bat.

**Figure 1:**
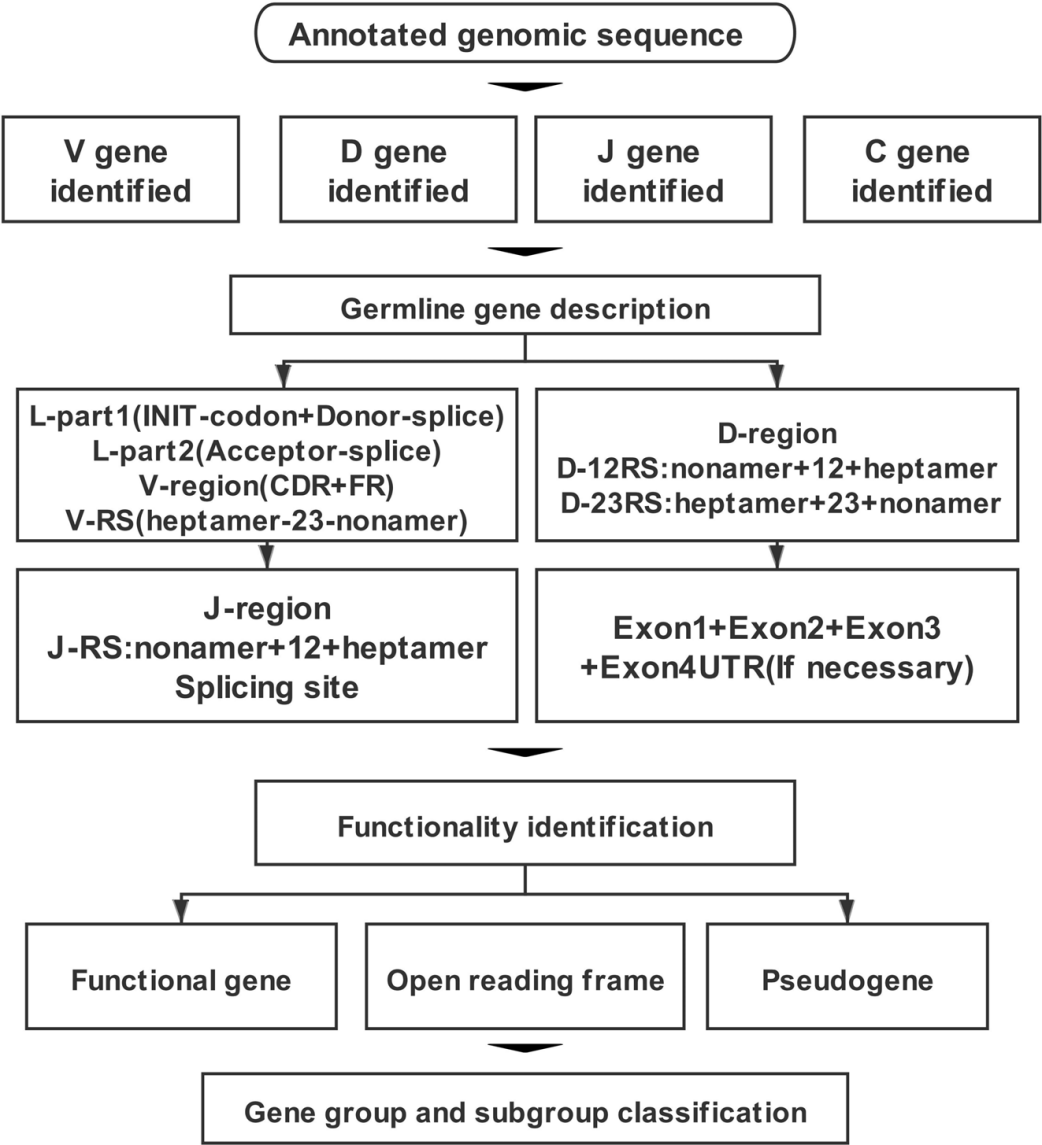
The annotation process of T-cell antigen receptor (TR) locus includes four parts: identification, description, functional determination, and family clustering of germline genes.

Annotation work was undertaken following the process outlined in **Figure 1**. The first step was to locate the TR locus in the chromosome. According to the gene information of mammalian TR loci recorded in the IMGT database, TRA/D loci are usually located in OR10G2 (Olfactory receptor family 10 subfamily G member 2) gene and DAD1 (Defender against cell death 1). TRG locus is located between AMPH (Amphiphysin) gene and STARD3NL (Related to steroidogenic acute regulatory protein D3-N-terminal like) gene. TRB locus is located between MOXD2 (Monooxygenase-beta-hydroxylase-like 2) and EPHB6 (Ephrin type-b receptor 6) genes.

The second step was the identification of germline genes. We analyzed sequences among the borne genes, e.g., V gene based on human and mouse patterns by the IgBLAST tool. For V gene of TRA/D locus, if the output result was TRAV/TRDV, we annotated it as V gene shared by TRA and TRD loci. Furthermore, the sequence was imported into Geneious Prime software, and the homology of the sequence was compared using the germline gene information recorded in the IMGT database, including the existing V, D, J, and C genes of humans, mice, pigs, and dogs. The third step was description of the germline genes. Here, four parts were described according to the types of germline genes.

Part (1): V gene is composed of three parts: L-Part+V-exon+V-RS. The L-part contains two segments, L-part1 and L-part2, with a part of intron between part1 and part2. L-part1 contains two conservative components: Init-codon and Donor-splice. L-part2 contains one conservative component: the Acceptor-splice. Part2 is next to the V-exon part, which is divided into six segments: FR1+CDR1+FR2+CDR2+FR3+CDR3. The three FR regions contain three conservative amino acid positions: 1st-Cys, Conserved-Trp, and 2nd-Cys. The last original part is 23 RSS: V-Heptamer+V-Spacers (23 bp)+V-Nonamer.

Part (2): The D gene consists of three parts: 5′D-RS+D-region+3′D-RS. 5′D-RS includes 5′D-Nonamer+5′D-Spacers (12 bp)+5′ Heptamer. The D-region usually consists of about 10- to 15-bp G-rich sequences. The 3′D-RS includes 3′D-Heptamer+3′D-Spacers (23 bp)+3′D-Nonamer.

Part (3): The J gene consists of two parts: J-RS+J-region. J-RS includes J-Nonamer+J-Spacer (12 bp)+J-Heptamer. There are two conserved components in the J-region: the [W,F]-[G,A]-X-G motif and splicing site.

Part (4): The composition of C gene is different due to its loci: TRAC and TRDC usually consist of four exons: Exon1+Exon2+Exon3+Exon4UTR. TRGC usually consists of three exons: Exon1+Exon2+Exon3. TRGC-Exon2 usually has multiple situations: Ex2A, Ex2B, Ex2C, Ex2R, and Ex2T. TRBC typically consists of four exons: Exon1+Exon2+Exon3+Exon4.

The fourth step was the functional identification of germline genes, for which the following guidelines were used: 1) 3′ RSS sequence of V gene (7–23–9), J gene 5′ end RSS sequence (9–12–7), 5′ end RSS sequence (9–12–7), and 3′ end RSS sequence (7–23–9) of D gene; 2) consideration of whether the 5′ end of V gene contains a leader sequence; 3) conserved acceptor and donor splicing site; 4) length of coding region; and 5) frameshift or stop codon of coding region. The final step was classification of group and subgroup of germline genes. For each identified and analyzed V gene, a phylogenetic evolution tree was constructed to name V gene uniformly based on existing species. If no homologous V gene was found, it was named according to its position in the loci. Each D, J, and C gene was named according to its physical location in its own cluster.

### 2.2 Comparison of the T-Cell Antigen Receptor Locus of *Rhinolophus ferrumequinum* and Other Mammals

The analysis results of TRA/D, TRG, and TRB loci were drawn into physical maps by Geneious Prime software according to the annotation of IMGT. Information about the four TR loci of each species in the IMGT database was compared. At the same time, the number of germline genes and subgroups among species was compared, and the nucleotide and amino acid composition of annotated genes were analyzed. With the use of a classical RSS sequence, the mutation number was counted, and a conservative analysis of 12/23 RSS sequences of V and J genes was carried out.

## 3 Results

### 3.1 TRA/TRD Locus

**Figure 2:**
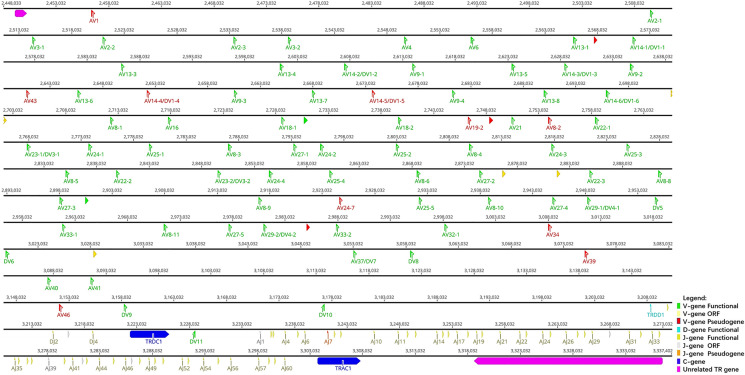
The schematic representation of the genomic organization of the bat TRA/D locus. The diagram shows the position of all the related and unrelated TRA/D genes according to nomenclature. The L-part and RSSs are not shown. The arrows indicate the transcriptional orientation of the genes. The number represents the specific location of the genes in chromosome 6 of *Rhinolophus ferrumequinum* (NC_046289.1).

Sequences analyzed included the following: 847 kb between OR10G2 and DAD1, 174 kb between AMPH and STARD3L, and 240 kb between MOXD2 and EPHB6. Using the whole genome assembly (GCA_004115265.3) of *R. ferrumequinum* recorded in the NCBI, we identified TRA/D locus (**Figure 2**). The result of the TRA/D locus annotation described below are summarized in **Table 1**. TRA/D locus with forward orientation was located on chromosome 6 (NC_046289); 5′ end borne gene-OR10G2 (2449106…2450211, GeneID: 117023609); and 3′ end borne gene-DAD1 (3318653…3336546, GeneID: 117024015). The total length of TRA locus from the first TRAV gene at the 5′ end to the last TRAC gene at the 3′ end was approximately 850 kb (2456031…330546), including 81 TRAV genes (classified into 34 groups), 60 TRAJ genes (classified into 60 groups), and one TRAC gene. TRA and TRD loci share a part of V gene, but TRD locus has its own D, J, and C genes. The total length of TRD locus from the first TRDV gene to the last reversed TRDV gene was approximately 660 kb (2571156…3228878), including 18 TRDV genes (classified into 11 families), four TRDJ genes (classified into four families), one TRDD gene, and one TRDC gene. The entire organization of TRA/D locus is as follows: Vα(55)-Vα/δ(10)-Vδ(1)-Vα(8)-Vδ(1)-Vα(2)-Vδ(1)-Vα(4)-Vδ(2)-Dδ(1)-Jδ(4)-Cδ(1)-Vδ(1)-Jα(60)-Cα(1). The structure of TRA/D loci in bats suggests that many TRAV and TRDV genes are located upstream of the loci. TRD loci share a part of V genes with TRA loci, and TRD loci have a complete D–J–C cluster, including one TRDD gene, four TRDJ genes, and one TRDC gene. There is a TRDV gene with opposite transcription direction downstream of TRDC gene, and finally a TRA locus J–C cluster, including 60 TRAJ genes and one TRAC gene. **Supplementary Table 2** provides detailed information for each annotated TRA/D locus germline gene in chromosome 6.

**Table 1:** Statistical analysis of TRA/D loci in humans (*Homo sapiens*), mice (*Mus musculus*), cows (*Bos taurus*), dogs (*Canis lupus familiaris*), cats (*Felis catus*), rhesus monkeys (*Macaca mulatta*), sheep (*Ovis aries*), rabbits (*Oryctolagus cuniculus*), and bats (*Rhinolophus ferrumequinum*).

Species	Chromosome orientation	Size (kb)	Number of TRAV/TRDV genes	Number of TRAJ/TRDJ genes	Number of TRDD genes	Number of TRAC/TRDC genes
*H. sapiens*	14 (FWD)	1,000	54/8	61/4	3	1/1
*M. musculus*	14 (FWD)	1,650	98/16	60/2	2	1/1
*B. taurus*	10 (REV)	3,330	183/39	60/4	9	1/1
*C. lupus familiaris*	8 (FWD)	760	58/5	59/4	2	1/1
*F. catus*	B3 (FWD)	830	63/10	64/5	2	1/1
*M. mulatta*	7 (FWD)	806	66/5	61/4	4	1/1
*O. aries*	7 (REV)	2,880	277/70	79/4	9	1/1
*O. cuniculus*	17 (FWD)	900	62/4	58/3	2	1/1
*R. ferrumequinum*	6 (FWD)	850	84/18	60/4	1	1/1

### 3.2 TRG Locus

**Figure 3:**
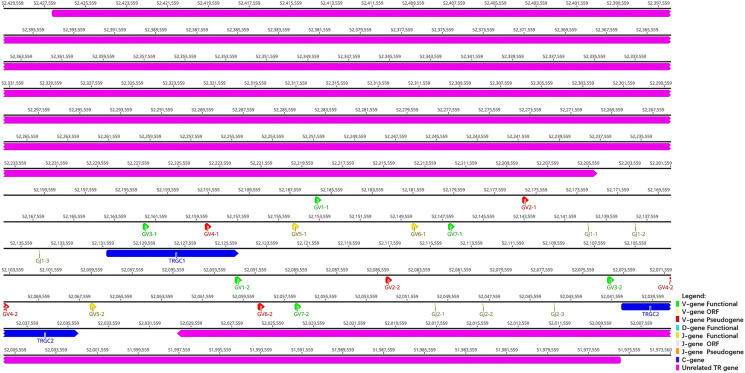
The schematic representation of the genomic organization of the bat TRG locus. The diagram shows the position of all the related and unrelated TRG genes according to nomenclature. The L-part and RSSs are not shown. The arrows indicate the transcriptional orientation of the genes. The number represents the specific location of the genes in chromosome 20 of *Rhinolophus ferrumequinum* (NC_046303.1).

TRG locus (**Figure 3**) is located on chromosome 20 (NC_046303) in reverse orientation: 5′ end borne gene-AMPH (52427198…52205147, GeneID: 117012456) and 3′ end borne gene-STARD3NL (52030211…51976009, GeneID: 117012446). The result of the TRG locus annotation described below are summarized in **Table 2**. The whole length of TRG locus from the first TRGV gene at 5′ end to the last TRGC gene at 3′ end is approximately 150 kb (52427198…51976009), including 14 TRGV genes (classified into seven families), six TRGJ genes (classified into three families), and two TRGC genes (classified into two families). The organization of TRG locus is as follows: Vγ(7)-Jγ(4)-Cγ(1)-Vγ(7)-Jγ(4)-Cγ(1). It suggests that TRG locus of *R. ferrumequinum* is composed of two V–J–C gene clusters, and each gene cluster is composed of the same number of genes, including seven V genes, four J genes, and two C genes. **Supplementary Table 3** provides detailed information for each annotated TRG locus germline gene in chromosome 20.

**Table 2:** Statistical analysis of TRG loci in humans (*Homo sapiens*), mice (*Mus musculus*), dogs (*Canis lupus familiaris*), cats (*Felis catus*), rhesus monkeys (*Macaca mulatta*), rabbits (*Oryctolagus cuniculus*), and bats (*Rhinolophus ferrumequinum*).

Species	Chromosome orientation	Size (kb)	Number of TRGV genes	Number of TRGJ genes	Number of TRGC
*H. sapiens*	7 (REV)	160	12–15	5	2
*M. musculus*	13 (FWD)	200	7	4	4
*C. lupus familiaris*	18 (FWD)	450	16	16	8
*F. catus*	A2 (FWD)	290	12	12	6
*M. mulatta*	3 (FWD)	145	12	5	2
*O. cuniculus*	10 (REV)	80	11	2	1
*R. ferrumequinum*	20 (REV)	150	14	6	2

### 3.3 TRB Locus

**Figure 4:**
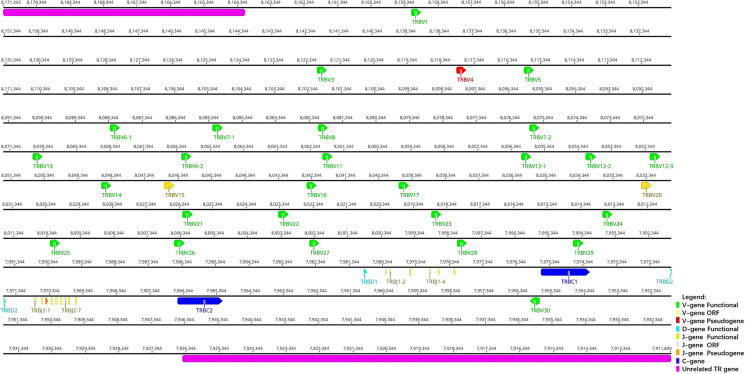
The schematic representation of the genomic organization of the bat TRB locus. The diagram shows the position of all the related and unrelated TRB genes according to nomenclature. The L-part and RSSs are not shown. The arrows indicate the transcriptional orientation of the genes. The number represents the specific location of the genes in chromosome 26 of *Rhinolophus ferrumequinum* (NC_046309.1).

TRB locus (**Figure 4**) is located on chromosome 26 (NC_046309) in reverse orientation: the 5′ end borne gene-MOXD2 (8164121 … 8171343, Gene ID: 117018015) and the 3′ end borne gene-EPHB6 (7911840 … 7926436, Gene ID: 117018107). The result of the TRB locus annotation described below are summarized in **Table 3**. The total length of TRB locus from the first TRBV gene at 5′ end to the last reverse TRBV gene at 3′ end is approximately 203 kb (8159483…7911840), including 29 TRBV genes (classified into 25 families), two TRBD genes (classified into two families), 15 TRBJ genes (classified into two families), and two TRBC genes. The entire organization of TRB locus is as follows: Vβ(28)-Dβ(1)-Jβ(6)-Cβ(1)-Dβ(1)-Jβ(9)-Cβ(1)-Vβ(1).

**Table 3:** Statistical analysis of TRB loci in humans (*Homo sapiens*), mice (*Mus musculus*), dogs (*Canis lupus familiaris*), cats (*Felis catus*), rhesus monkeys (*Macaca mulatta*), rabbits (*Oryctolagus cuniculus*), and bats (*Rhinolophus ferrumequinum*).

Species	Chromosome orientation	Size (kb)	Number of TRBV genes	Number of TRBD genes	Number of TRBJ genes	Number of TRBC
*H. sapiens*	7 (FWD)	620	65–68	2	14	2
*Sus scrofa*	18 (REV)	407	38	3	20	3
*C. lupus familiaris*	16 (REV)	271	36	2	12	2
*F. catus*	A2 (FWD)	302	33	2	12	2
*M. mulatta*	3 (FWD)	736	77	2	14	2
*Ovis aries*	4 (FWD)	506	94	3	19	3
*R. ferrumequinum*	26 (REV)	203	29	2	15	2

Data available in international ImMunoGeneTics information system (IMGT) Repertoire (IG and TR) http://imgt.org/IMGTrepertoire/>Locus and genes>Locus description>Locus descriptions>TRA/D, TRG, TRB>Human, Mouse, Cow, Dog, Cat, Rhesus monkey, Sheep, Rabbit, Pig.

The structure of TRB locus of *R. ferrumequinum* indicates that upstream is a V cluster composed of 28 TRBV genes, and downstream are two complete D–J–C gene clusters. The first cluster contains: one D gene, six J genes, and one C gene. The second cluster contains one D gene, nine J genes, and one C gene. There is a TRBV gene with the opposite transcription direction downstream of the second TRBC gene. Detailed information about each annotated TRB locus germline gene in chromosome 26 is given in **Supplementary Table 4**.

### 3.4 Classification of Germline Gene in Four T-Cell Antigen Receptor Locus of *Rhinolophus ferrumequinum*

#### 3.4.1 Classification and Phylogenetic Analysis of V Genes

1) A total of 81 TRAV genes were found in TRA loci, and the nucleotide similarity of these 81 genes ranged from 25.1% to 98.9%. With the use of 75% as a standard for nucleotide homology where genes belong to the same group, it was divided into 31 groups. To analyze the classification and affiliation between annotated V genes of bats and other species, we selected V gene sequences of primate representative species (humans), carnivorous representative species (dogs), and artiodactyl representative species (cattle) from the IMGT database to construct phylogenetic trees. There were two criteria for selecting genes: one was to select only potential functional genes and in-frame pseudogenes, and the other was to select only one gene for each subgroup.

**Figure 5:**
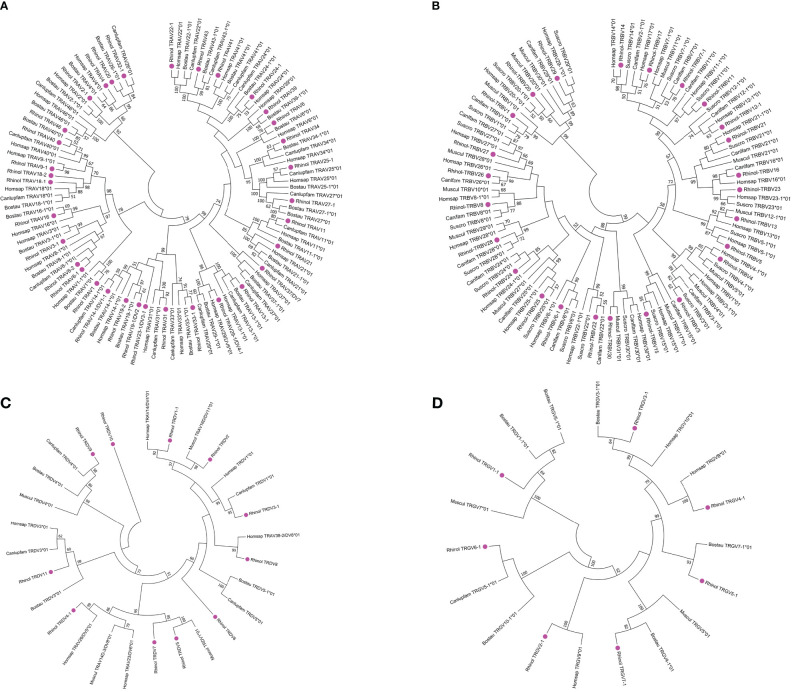
Phylogenetic analysis of TRAV **(A)**, TRBV **(B)**, TRDV **(C)**, TRGV **(D)** genes. Unrooted trees were constructed using the neighbor-joining method based on V-REGION nucleotide sequences of bats, dogs, humans, mice, and cows. The percentage of the nodes in 1,000 bootstrap replicates are show on the branches. Bat genes are labeled in pink. The international ImMunoGeneTics information system (IMGT) standardized abbreviation for taxon is used: six letters for species (*Rhinolophus ferrumequinum*, Rhinol; *Homo sapiens*, Homspa; *Mus musculus*, Muscul; *Bos taurus*, Bostau) and nine letters for subspecies (Canlupfam).

After all the selected V genes were compared by ClustalW method, MEGA7 was used for the neighbor-joining (N-J) method of rootless tree construction. The phylogenetic tree constructed using 31 bat TRAV genes is shown in **Figure 5A**. The results reflect the homology of the annotated bat TRAV genes with other species. We named the bat TRAV genes according to the comparison results. There are three groups—TRAV8, TRAV18, and TRAV19—of which TRAV8-2 gene and the rest of TRAV8 members contribute only about 65% identity. However, in the evolutionary branch, we found that TRAV8-2 and TRAV8-1 clustered to the same branch. As TRAV8-2 is a pseudogene, we incorporated it into TRAV8 group. The same situation occurred with the identity between TRAV18-1 and TRAV18-2 of 73.8%, and TRAV19-1 and TRAV19-2 at 69.6%. Of the 31 families, 27 can form branches with human and cattle genes at the same time. Genes that failed to find homology were named according to their positions in the locus. There are 16 multigene groups in TRAV, and six groups have been significantly amplified, including TRAV8, TRAV13, TRAV14, TRAV24, TRAV25, and TRAV27 (each family has five members). Ten groups are composed of two to four members, namely, TRAV2, TRAV3, TRAV9, TRAV18, TRAV19, TRAV22, TRAV23, TRAV29, TRAV32, and TRAV33; and the remaining 20 are single gene groups.

2) There are 18 TRDV genes in TRD locus, and the nucleotide identity is between 27% and 98.6%. The 18 TRDV genes are divided into 11 families, including TRDV5, TRDV6, TRDV7, TRDV8, TRDV9, TRDV10, TRDV11, and single gene groups. The phylogenetic tree constructed by the 11 TRDV genes of bats with humans, dogs, and cattle is shown in **Figure 5C**. Except for TRDV10 family, all the bat groups were homologous with the other three species. Of the 18 TRDV genes, only two shared with TRA loci were classified as pseudogenes, and the remaining 16 were functional genes.

3) TRG locus contains 14 TRGV genes with nucleotide similarity ranging from 33% to 99%. Because TRG locus of the bat is composed of two identical V–J–C gene clusters, the 14 TRGV genes are divided into seven groups with nucleotide identity ranging from 94.6% to 99.0%. A phylogenetic tree was constructed with the annotated TRDV genes of the bat and TRDV genes of humans, dogs, cattle, and mice (**Figure 5D**). All TRGV genes of the bat were homologous with the other four species. The 14 TRGV genes have a high proportion of pseudogenes. Five of 14 were classified as pseudogenes, three as ORFs, and six as functional genes. Because the recorded data of TRDV and TRGV genes between species are limited and the uniformity is not high, they were named according to position in the locus.

4) TRB locus contains 29 TRBV genes with nucleotide identity ranging from 33.7% to 94.2%. The 29 TRBV genes were divided into 25 groups. The phylogenetic trees (**Figure 5B**) constructed for humans, mice, dogs, and pigs showed that all the annotated TRBV genes of the bat were homologous with the other species. Human TRBV1 could not cluster with TRBV1 group of cattle, mice, pigs, and bats but was on the branch of TRBV4. There are three multigene groups, with TRBV5 and TRBV6 having two gene members and TRBV12 having three gene members. The 29 TRBV genes have one pseudogene and two ORFs, and the remaining 26 are classified as functional genes. The descriptions of pseudogenes and ORFs of all germline genes of the TR locus of bats are shown in **Supplementary Table 5**.

**Figure 6:**
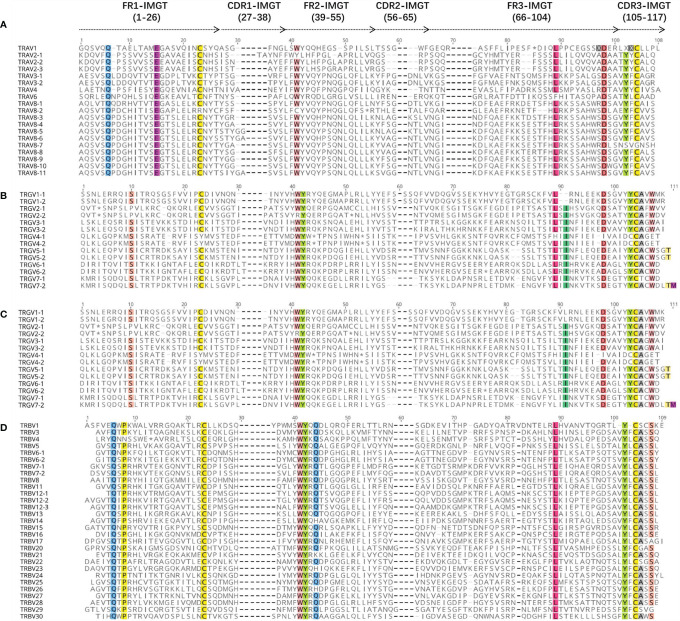
The international ImMunoGeneTics information system (IMGT) Protein display of the bat TRAV **(A)**, TRDV **(B)**, TRGV **(C)**, and TRBV **(D)** genes. TRAV genes are only shown at TRAV1 to TRAV10. The description of the FR-IMGT and CDR-IMGT is according to IMGT unique numbering for V-region. The four conserved amino acid of the V-domain (1st-CYS 23, CONSERVED-TRP 41, hydrophobic AA 89, 2nd-CYS 104) are indicated in colors.

The amino acid sequences of some TRAV genes (81 TRAV genes in **Supplementary Figure 1**) and all TRDV, TRGV, and TRBV genes annotated for bats were aligned according to the unique number of the IMGT V-region (**Figure 6**), to ensure maximum homology.

#### 3.4.2 Analysis of D Gene and J Gene

**Figure 7:**
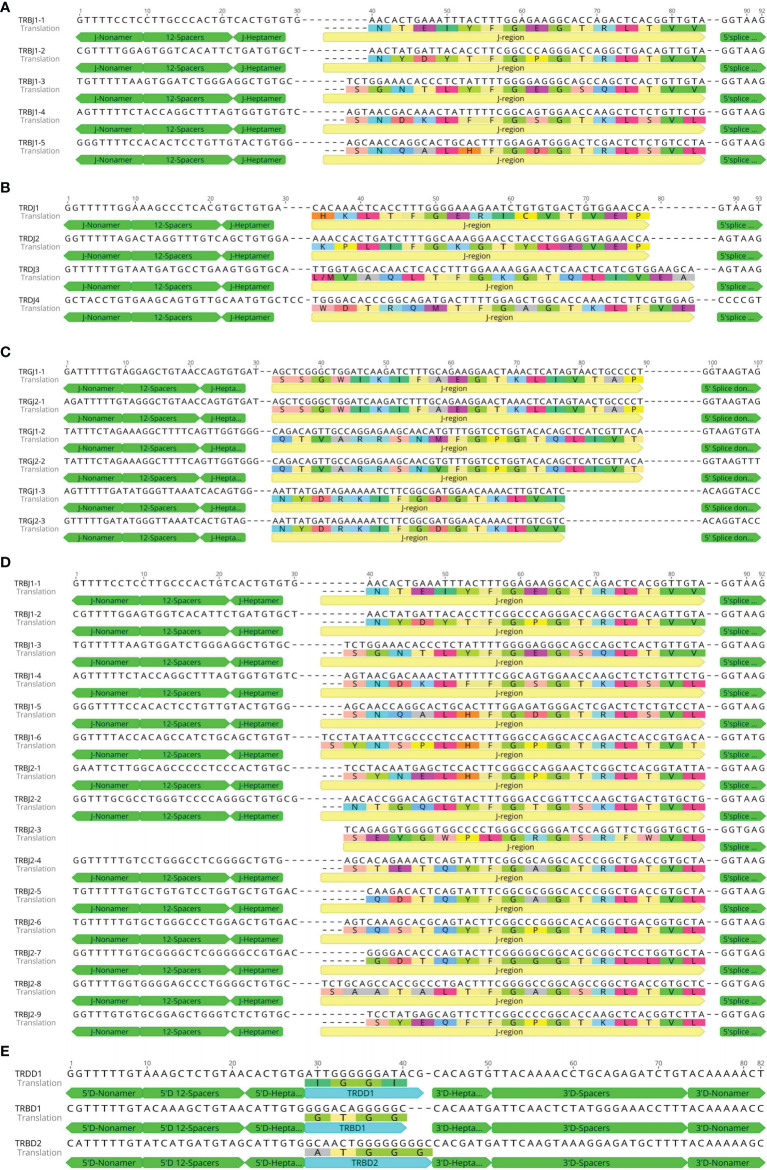
Nucleotide and deduced amino acid sequences of the bat TRAJ **(A)**, TRDJ **(B)**, TRGJ **(C)**, TRBJ **(D)**, TRBD, and TRBD **(E)** genes. The heptamer, nonamer, and splice donor are labeled. The numbering adopted for the gene classification is reported on the left of each gene.

According to the naming rules stipulated by IMGT, J genes are classified by C genes and numbered according to the positions in their respective (D)–J–C gene clusters. The length of J gene is 51–66 bp, and the typical [W, F]-[G, A]-X-G motif is retained, determining the function of J gene. There are 12 RSS sequences at the 5′ end and donor splicing sites at the 3′ end of each J gene. We annotated a total of 60 TRAJ genes with nucleotide similarity between 22.2% and 71.9%. According to nucleotide homology, they can be divided into 60 families. The nucleotide and amino acid-deduced sequences of TRAJ are shown in **Figure 7A** (**Supplementary Figure 2** for 60 TRAJs). Only TRAJ1 and TRAJ7 do not contain the conserved [W, F]-[G, A]-XG motif. Of the 60 TRAJ genes, TRAJ1, TRAJ30, TRAJ39, TRAJ42, and TRAJ47 were classified as ORFs; TRAJ7 was classified as a pseudogene; and the remaining 54 were functional genes.

A total of four TRDJ genes were annotated with nucleotide similarity between 48.9% and 66.7%, divided into four families. The nucleotide and amino acid-deduced sequences of TRDJ are shown in **Figure 7B**. Three TRDJ genes contained the conserved FGXG motif and were therefore classified as functional genes. Only TRDJ3 was classified as an ORF. A total of six TRGJ genes were annotated with nucleotide similarity between 53.1% and 100%, which can be divided into two families. The nucleotide and amino acid-deduced sequence are shown in **Figure 7C**. The similarity between TRGJ1-1 and TRGJ2-1 is 100%, TRGJ1-2 and TRGJ2-2 is 98.3%, and TRGJ1-3 and TRGJ2-3 is 98%. The six TRGJ genes all contain the conserved F-[G, A]-XG motif and RSS sequences and thus were classified as functional genes. A total of 15 TRBJ genes were annotated. As TRB locus contains two D–J–C clusters, the 15 TRBJ genes were divided into two families, with nucleotide similarity ranging from 33.3% to 91.1%. The similarity of TRBJ-4 with TRBJ-5 and TRBJ-6 reached 91.1% and 84.4%, respectively. The nucleotide and amino acid-deduced sequences are shown in **Figure 7D**. Only one D gene was found in TRD locus. The nucleotide and amino acid-deduced sequence of TRDD1 gene are shown in **Figure 7E**, consisting of a 14-bp G-rich fragment, and very conservative RSS sequences upstream and downstream. Two D genes were found in TRBV locus. The nucleotide and amino acid-deduced sequences of TRBD1 and TRBD2 genes are shown in **Figure 7E**. The two TRBD genes are composed of 12- and 15-bp G-rich fragments.

#### 3.4.3 Analysis of C Gene

**Figure 8:**
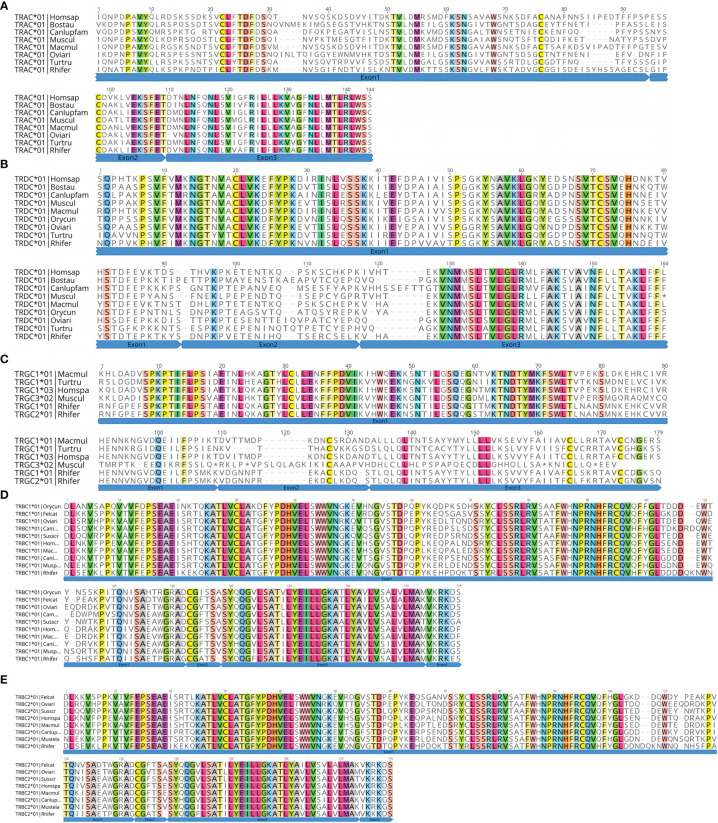
Deduced amino acid sequences of the bat TRAC **(A)**, TRDC **(B)**, TRGC **(C)**, TRBC1 **(D)**, and TRBC2 genes **(E)**. *Rhinolophus ferrumequinum*, Rhifer; *Homo sapiens*, Homspa; *Mus musculus*, Muscul; *Bos taurus*, Bostau; *Camelus dromedarius*, Camdro; *Tursiops truncatus*, Turtru; *Oryctolagus cuniculus*, Orycun; *Sus scrofa*, Susscr; *Ovis aries*, Oviari; *Macaca mulatta*, Macmul; *Felis catus*, Felcat; *Mustela putorius furo*, Musputfur; *Canis lupus familiaris*, Canlupfam.

At the genome level, each C gene is composed of multiple exons. EX1 encodes a constant region, part of the 5′ ends of EX2; EX3 encodes a connecting region, part of the 3′ end of EX3. The first codon of EX4 encodes a transmembrane region, and the rest of EX4 encodes a cytoplasmic region. The fourth exon of TRAC and TRDC is usually untranslated Exon4UTR, while TRGC does not have the fourth exon. Only TRBC usually contains a complete amino acid sequence of the four exons. Exon1, Exon2, and Exon3 have the same size, but different species have different introns. We analyzed the amino acid sequence and intron/exon structure of the annotated C gene of the bat with each other species (**Figure 8** and **Supplementary Figure 3**). We found one TRAC gene and one TRDC gene in TRA/D locus. TRAC1 gene of bats encodes 136 amino acids. The amino acid similarity of TRAC gene between species is 47.1%–81.4%. The highest similarity between TRAC genes of bats and rabbits is 54.5%. Exon1 of TRAC varies from species to species, while Exon2 and Exon3 are highly conserved. TRDC1 gene of bats encodes 142 amino acids, and the amino acid sequence similarity of TRDC gene of each species ranges from 64.1% to 86.35. The similarity between TRDC gene of bats and rhesus monkeys reaches 72.6%. Except for Exon4UTR, the remaining three exons are highly conserved. There are two TRGC genes in TRG locus of the bat, both of which are encoded by 163 amino acids. Because TRGC has multiple Exon2 structures, the intron length and structure of TRGC genes in different species vary. However, we only matched one Exon2 among the two TRGC genes of the bat. There are two TRBC genes in TRB locus of the bat, both of which are encoded by 175 amino acids and consist of four complete exons. The similarity of amino acid sequences among TRBC1 species is between 72.5% and 92.6%, and the similarity of TRBC2 is between 71.3% and 93.8%.

### 3.5 Germline Gene Recombination Signal Sequence Analysis

**Figure 9:**
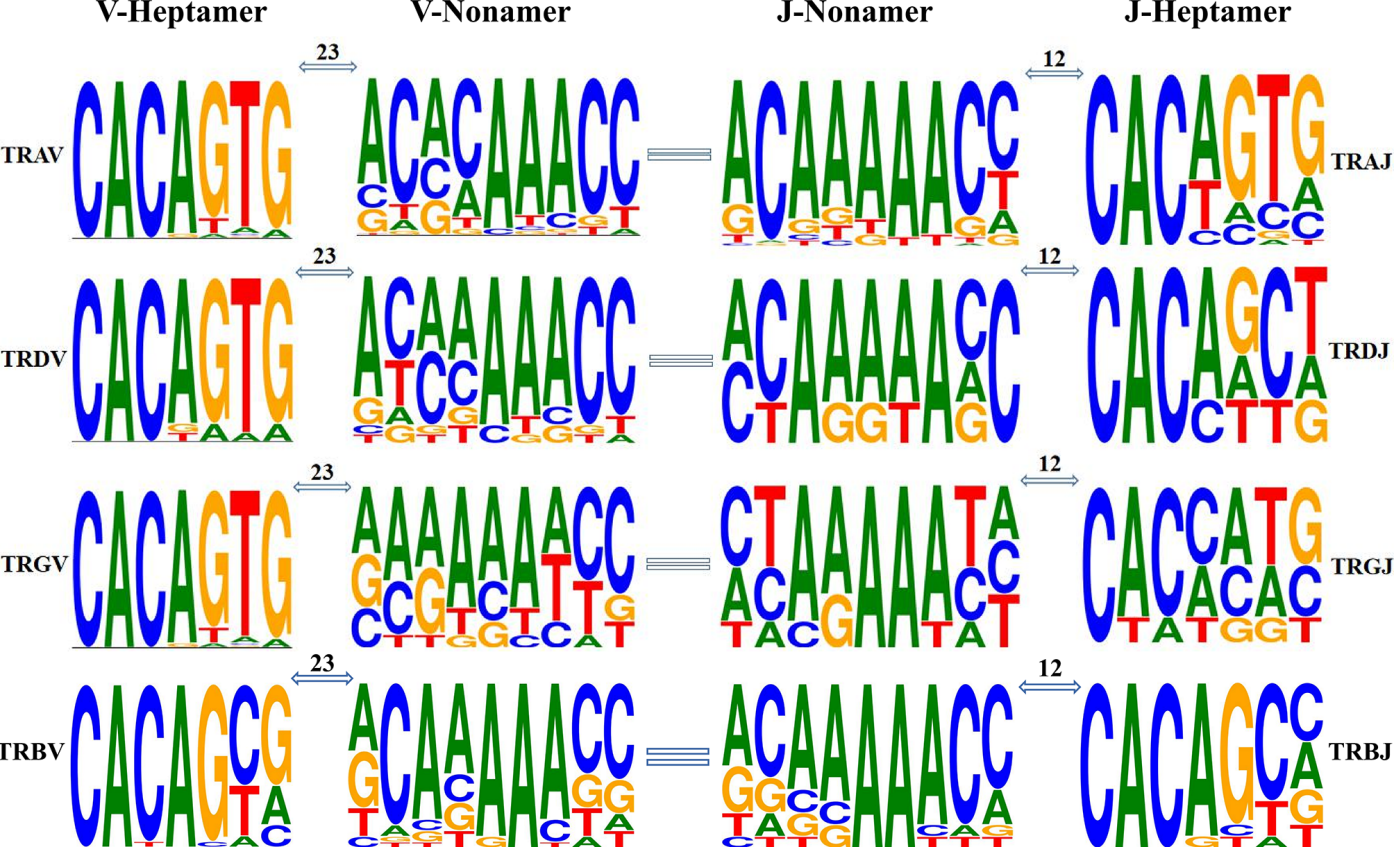
Position weight matrixes of recombination signal sequences of T-cell receptor V and J genes. The height of symbols indicates the relative frequency of each nucleotide at that position.

Using IMGT guidelines, we statistically analyzed the RSS sequences judged as germline genes (**Figure 9**). We used the classic 7-mer-CACAGTG (heptamer) and 9-mer-ACAAAAACC (nonamer) as motifs. The number of mutations in the RSS sequence was analyzed: in TRA/D locus, seven of the 81 TRAV genes did not have the 23-RSS sequence, and the number of mutations in the 74 RSS sequences was between zero and nine. The 3′ ends of 18 TRDV genes all have 23-RSS sequences, and the number of mutations in the 18 RSS sequences was between zero and nine; the number of mutations in 15 sequences was less than six; the number of mutations in two sequences was eight; and the number of mutations in one sequence was nine. Of the 60 TRAJ genes, five had no 12-RSS sequence at the 3′ end, and the number of mutations in fifty-five 12-RSS sequences was between zero and seven, of which the number of mutations in 54 sequences was less than six and the number of mutations in one sequence was seven. In TRG locus, two out of 14 TRGV genes did not have 23-RSS sequence, and the number of mutations in the 23-RSS sequence of 12 TRGV genes was between zero and nine, of which 11 have fewer than seven mutations. The number of mutations in one sequence was nine. The six TRGJ genes all have 12-RSS sequences at the 3′ end, and the number of mutations in the 12-RSS sequences of the six TRGJ genes ranged from zero to nine, of which the number of mutations in three genes was less than five and the number of mutations in three genes was nine. In TRB loci, 29 TRBV genes all had 23-RSS sequences, and the number of mutations in 23-RSS sequences of TRBV genes was between one and six, of which only one mutation number was six, and a total of 20 mutations were concentrated in three to four mutations. Only one of the 15 TRBJ genes does not have the 12-RSS sequence, and 14 genes had between two and six mutations in 12 RSS, mainly between three and five. It can be seen that the RSS sequence of TRB locus is more conservative than the other loci.

The heptamer of all the V genes and the poly-A tract of V gene nonamer are highly conserved. The nonamer of TRAV and TRDV has a high degree of diversity in the first four nucleotides, while the whole nonamer of TRGV has high diversity. The diversity of the RSS sequence is more obvious in J gene, although the poly-A tract of J gene nonamer is conservative. The first two nucleotides and the last two of J gene nonamers are diversified, more obvious in the heptamer of J gene. However, the bases (CAC) of the first three nucleotides and the last four nucleotides of J gene heptamer are highly diversified.

## 4 Discussion

The function of vertebrate T/B cells depends on TCR/BCR, and the TR locus analysis of species is of great significance in understanding immune responses. Information about the four TR loci—TRA/TRD, TRG, and TRB—of many species is included in the IMGT database, and the TR loci of 12 species, including humans, macaques, mice, pigs, cows, sheep, dogs, cats, dolphins, ferrets, rabbits, and zebrafish, have been completely annotated and described (http://www.imgt.org/IMGTrepertoire/LocusGenes/#C). Previous studies have annotated and analyzed one or two loci of a single species. However, for the first time, we annotated the four loci of *R. ferrumequinum*, the greater horseshoe bat, directly through the whole genome for the distribution characteristics, sequence characteristics, and conservative RSS of germline genes. This work generates suggestions and reference methods for new species with unresolved TR/IG loci. We also believe that high-quality TR locus annotation is the key to the subsequent analysis of the TCR/BCR repertoire of *R. ferrumequinum*.

Gene replication is the basis of the evolution of antigen-specific receptors, leading to the formation of independent IG/TR sites (34, 35). As mammalian TRD loci are embedded in TRA loci, the length of this locus is the longest of the TR loci. Still, despite this, the structure has not changed much (36), and the amplification events in the genome are part of the reasons for the differences. In TRA/D locus, the most obvious replication events occurred in artiodactyl cattle (3,330 kb) and sheep (2,880 kb). In terms of the structural characteristics of the loci, TRA/D locus of *R. ferrumequinum* also maintained the conservative structure of mammals. Only primate macaques (806 kb), carnivorous cats (830 kb), and dogs (760 kb) have shorter TRA/D loci than *R. ferrumequinum* (850 kb).

In contrast, although the coverage of TRG loci is the shortest, the situation among species may be more complicated. Many species have detected the existence of two TRG loci. For example, Atlantic salmon have two loci, TRG1 and TRG2, although the expression of TRG2 cannot be detected (37). In addition, the study of sheep revealed at least two TRG loci on chromosome 4, and expression analysis showed that both have functions (38, 39). The second TRG locus was found in cattle, buffalo, and goats by the same method (40, 41). In contrast, TRG locus structure of *R. ferrumequinum* is relatively simple and consists of two V–J–C gene clusters with an almost identical composition. The dog’s TRG locus has undergone repeated replication, resulting in eight V–J–C gene clusters (42). The mammalian TRB locus is probably the most conservative, with multiple V genes in the upstream, multiple D–J–C clusters in the downstream, and replication events occurring among D–J–C gene clusters. In the early analysis of chicken expression level, only one D–J–C cluster was found (43). Primate humans, macaques, rodent mice, carnivorous cats, dogs, and *R. ferrumequinum* all contain two D–J–C clusters. Artiodactyla, including pigs and sheep, represent three D–J–C gene clusters. We found that TRBJ gene of the second D–J–C cluster and the third cluster of pigs and sheep is highly homologous. The replication event is likely to occur in the second gene cluster, and there are four D–J–C clusters downstream of TRB locus of the short-tailed opossum (12). In addition, TRB loci of primate humans (620 kb) and rhesus monkeys (736 kb) have the largest span, and the number of embryonic genes is only slightly less than that of sheep (506 kb). It is worth noting that a TRDV gene and a TRBV gene with opposite transcription directions are arranged downstream of TRDC and TRBC in mammals, and these two reverse-transcribed V genes are expressed in both humans and mice.

The 81 TRAV genes of *R. ferrumequinum* can be classified into 31 subgroups, of which 14 subgroups are multigene families, and obvious amplification occurs in TRAV8 (11 members), TRAV13 (eight members), TRAV14 (six members), and TRAV24 (seven members). Although 54 human TRAVs can be classified into 44 subgroups, there are only seven multigene groups, and only TRAV8 has more than three members. The most significant is the 183 TRAV of cattle, classified into 42 subgroups of which 34 are multigene groups; and TRAV22, TRAV23, TRAV25, and TRAV26 all have obvious amplification (44). In the 18 TRDVs of *R. ferrumequinum*, aside from TRDV1 (six members), TRDV3 (two members), and TRDV4 (two members), all other families are single-gene families. The amplification of TRA/D locus is particularly remarkable in artiodactyls. The number of TRDV1 families in cattle and sheep is 50 and 66, respectively, while TRDV1 has only 13 members in humans. The 29 TRBV genes of *R. ferrumequinum* can be classified into 25 families, and only TRBV6 (two members), TRBV7 (two members), and TRBV12 (three members) are multigene subgroups. According to the statistics of homologous groups of *R. ferrumequinum* (**Supplementary Table 6**), the amplification of events tends to be random. TRAV24 and TRAV25 of the greater horseshoe bat and cattle are polygenic families, but TRAV24 and TRAV25 of humans and dogs are monogenic families. Similarly, TRBV6s of the greater horseshoe bat and human are polygenic families, but TRBV6s of the dog and pig are monogenic (16). TRAV24 and TRAV25 of *R. ferrumequinum* and cattle are obviously polygenic groups, but for humans and dogs, they are monogenic groups. Similarly, TRBV6 of the horse-headed bat and human are polygenic families, but for dogs and pigs, they are monogenic (16).

We counted the CDR region length (**Supplementary Table 7**) of the homologous TRAV and TRBV genes of different species and concluded that the length of TRBV gene subgroup is conservative among species. Only the lengths of TRBV1 and TRBV17 differ between human beings and bats. However, the CDR region length of TRAV varies greatly among the four species, even though the loss of some statistical information and gene families has affected our analysis. Early researchers put forward an evolutionary model of IG and TCR genes, termed birth-and-death evolution. This evolutionary model explained the emergence of most polygenic families and that some genes become pseudogenes due to extensive mutations. Family amplification occurs simultaneously as mutations and deletions occur between families, a compensation mechanism to maintain diversity. The internal replication of the genome has become a routine event, and the birth of most germline genes comes from the replication of intergenomic fragments. This birth depends more on replicating existing genes than on the formation of new subgroups (45–47).

Birth of genes is accompanied by death because some genes always become pseudogenes because of mutations or frameshifting (45). Sixteen percent of 81 TRAVs, 11% of 18 TRDV genes, 35% of 14 TRGVs, and 3% of 29 TRBVs belong to pseudogenes. Correspondingly, the proportion of TRBV pseudogenes in humans, dogs, and pigs is 19%, 43%, and 31.5%, respectively. TRA/D locus of cattle shows not only a high “birth rate” but also a high death rate. The proportion of TRAV pseudogenes in humans, mice, and cattle is 19%, 16.5%, and 37.2%, respectively (8). Although there is no diversity in cattle, in contrast, 81 TRAV/DV genes of *R. ferrumequinum* show a larger and more stable germline gene pool than 54 TRAV/DV genes of human beings, similar to IGVH3 gene family of the small brown bat. There may be more preliminary discoveries of combination diversity (28).

We need to evaluate the diversity of the TCR or BCR receptor repertoire of a species. First, this is important in terms of the number of germline genes, which directly affects the diversity of rearrangement and considers the addition and mutation of nucleotides. In previous studies, somatic hypermutation occurred only in B cells of higher vertebrates to produce high-affinity antibodies, which are very rare in T cells. We counted all species in the IMGT database, including our annotated germline gene pool of *R. ferrumequinum*. Unlike TRAV gene, most mammals have approximately 60 TRAJ genes. It is worth noting that artiodactyls such as sheep and cattle have many replication events in TRA/D locus and TRA/D V gene. The number of TRAJ genes in sheep is 79, but the number of artiodactyl cattle has not increased significantly. The researchers speculated that the 19 TRAJ complementary genes found in sheep might result from replication from TRAJ29 to TRAJ39 due to sequencing errors or amplification (44). The statistical results for D gene are similar. Only cattle and sheep have nine TRAD genes, and surprisingly, there is only one D gene in *R. ferrumequinum*, the only known species with just one TRDD gene at present.

Another feature of TRG loci is the number of TRGC and the diversity of corresponding protein structures. The size of TRGC gene is usually different, caused by the different exon numbers and intron lengths of the coding junction region—evident even among the same species. TRGC2, TRGC3, and TRGC5 of salmon are divided into three exons (EX1, EX2, and EX3), while TRGC1 has two EX2s (37). TRGC1 and TRGC5 of sheep have only one EX2(A), TRGC3 has two EX2(A and C), and TRGC6 has three EX2(A, B, and C) (48). TRGC5 of cattle has only one EX2, but TRGC3 and TRGC7 have two EX2, and TRGC1, TRGC2, TRGC4, and TRGC6 all have three EX2s (49). TRGC1 and TRGC2 of dromedaries also contain three EX2s (50, 51). TRGC2, TRGC3, and TRGC4 of dogs have two EX2s, while TRGC1, TRGC6, TRGC7, and TRGC8 have only one EX2. All monotremes and marsupials lack the second cystamine, necessary for forming intra-chain disulfide bonds, clearly caused by independent mutations (52).

We made a conservative analysis of the RSS sequences of germline genes, but we did not find non-classical RSS before and after V and J genes without RSS sequences. For example, if the spacer was 12 ± 1 bp or 23 ± 1 bp, it would not affect further analysis because the effective recombination only occurred between the 12-bp RSS and the 23-bp RSS (53). RSS sequence-conserved sites of the four loci of *R. ferrumequinum* are all related. Whether they are heptamer or nonamer, the first four nucleotides of the conserved heptamer sites are CACA, while the conserved site of the nonamer is usually the poly-A tract in the sequence. Because the first three nucleotides of heptamers play an important role in the recombination process, the differences in these positions may affect the recombination of genes. However, at present, we still do not know how—or how much—it will affect the rearrangement if we have atypical RSS before and after germline genes (54).

## 5 Conclusion

The bat is a special type of mammal that carries many virulent viruses but is not harmed by them. The mechanism of the bat’s viral resistance remains poorly understood because of difficulty obtaining samples and a general lack of relevant research (31). In this paper, the four TR loci of the greater horseshoe bat were annotated completely at the genome level. The structural differences of TR loci between other species and *R. ferrumequinum* were statistically analyzed, and differences in germline gene composition between species were discussed. Generally speaking, the four TCR loci of *R. ferrumequinum* are highly conserved compared with other mammals. Homologous genes defined by phylogeny may not participate in the rearrangement process, e.g., homologous genes of TRAV and TRDV, since it is necessary to analyze bat receptor repertoire data through specific primers. We hope this article could provide a basis for the study of the immune mechanism of bats.

## Data Availability Statement

Publicly available datasets were analyzed in this study. This data can be found here: https://www.ncbi.nlm.nih.gov/Taxonomy/Browser/wwwtax.cgi?mode=Info&id=59479.

## Ethics Statement

Ethical review and approval was not required for the animal study because it is one genome analysis.

## Author Contributions

XY and LM designed this research. HZ and XY wrote this manuscript. HZ and LL analyzed the data. All authors contributed to the article and approved the submitted version.

## Funding

The National Natural Science Foundation of China (31860257) and Guizhou Provincial High-level Innovative Talents Project [No. (2018) 5637] funded this study.

## Conflict of Interest

The authors declare that the research was conducted in the absence of any commercial or financial relationships that could be construed as a potential conflict of interest.

## Publisher’s Note

All claims expressed in this article are solely those of the authors and do not necessarily represent those of their affiliated organizations, or those of the publisher, the editors and the reviewers. Any product that may be evaluated in this article, or claim that may be made by its manufacturer, is not guaranteed or endorsed by the publisher.
